# The Prevalence and Characteristics of Fibromyalgia in the 2012 National Health Interview Survey

**DOI:** 10.1371/journal.pone.0138024

**Published:** 2015-09-17

**Authors:** Brian Walitt, Richard L. Nahin, Robert S. Katz, Martin J. Bergman, Frederick Wolfe

**Affiliations:** 1 National Center for Complementary and Integrative Health, National Institutes of Health, Bethesda, Maryland, United States of America; 2 Rush University Medical Center, Chicago, IL, United States of America; 3 Drexel University College of Medicine, Philadelphia, PA, United States of America; 4 National Data Bank for Rheumatic Diseases, Wichita, KS, United States of America; University of Sevilla, SPAIN

## Abstract

**Background:**

Most knowledge of fibromyalgia comes from the clinical setting, where healthcare-seeking behavior and selection issues influence study results. The characteristics of fibromyalgia in the general population have not been studied in detail.

**Methods:**

We developed and tested surrogate study specific criteria for fibromyalgia in rheumatology practices using variables from the US National Health Interview Survey (NHIS) and the modification (for surveys) of the 2010 American College of Rheumatology (ACR) preliminary fibromyalgia criteria. The surrogate criteria were applied to the 2012 NHIS and identified persons who satisfied criteria from symptom data. The NHIS weighted sample of 8446 persons represents 225.7 million US adults.

**Results:**

Fibromyalgia was identified in 1.75% (95% CI 1.42, 2.07), or 3.94 million persons. However, 73% of identified cases self-reported a physician’s diagnosis other than fibromyalgia. Identified cases had high levels of self-reported pain, non-pain symptoms, comorbidity, psychological distress, medical costs, Social Security and work disability. Caseness was associated with gender, education, ethnicity, citizenship and unhealthy behaviors. Demographics, behaviors, and comorbidity were predictive of case status. Examination of the surrogate polysymptomatic distress scale (PSD) of the 2010 ACR criteria found fibromyalgia symptoms extending through the full length of the scale.

**Conclusions:**

Persons identified with criteria-based fibromyalgia have severe symptoms, but most (73%) have not received a clinical diagnosis of fibromyalgia. The association of fibromyalgia-like symptoms over the full length of the PSD scale with physiological as well as mental stressors suggests PSD may be a universal response variable rather than one restricted to fibromyalgia.

## Introduction

Fibromyalgia is a medical diagnosis used to describe the diminished quality of life related to generalized body pains and physical and psychological symptoms that occurs in the absence of a clear pathologic cause. To be diagnosed with fibromyalgia requires that symptomatic persons seek health care from clinicians and that those clinicians interpret the described symptoms as being fibromyalgia. A person cannot have a fibromyalgia diagnosis unless they took the effort to see a clinician who is willing to make that diagnosis. For this reason, the clinical diagnosis of fibromyalgia is necessarily confounded by health care seeking behavior and clinical selection. Our current understanding of the epidemiology of fibromyalgia is primarily derived from research studies taking place in clinical settings. To date, fibromyalgia studies have not considered symptomatic persons in the population that have not been diagnosed. Being able to approximate fibromyalgia symptoms in a large, representative population outside the context of the clinical setting can provide new insight into the illness and its nature.

The development of the 2011 modification for survey research [1] of the American College of Rheumatology (ACR) Preliminary Diagnostic Criteria for Fibromyalgia [2] has enabled investigators to approximate both fibromyalgia diagnosis and severity outside of the clinical setting. As it does not require a physician examination, the modified 2011 criteria, can be used to determine a “calculated prevalence” of fibromyalgia in a given population. We define calculated prevalence as the prevalence of fibromyalgia if everyone who could satisfy the modified (research) fibromyalgia criteria were included in the numerator, whether or not they had received a diagnosis of fibromyalgia from a physician. In this way, fibromyalgia can be studied in a way that is not confounded by health care seeking and clinical selection issues.

To obtain information about fibromyalgia and fibromyalgia diagnosis in the general population, we developed surrogate fibromyalgia criteria from variables available in the 2012 National Health Interview Survey (NHIS), [3] and identified persons who satisfied them from symptom data collected in the survey. The National Health Interview Survey (NHIS) is a multi-purpose health survey conducted by the National Center for Health Statistics (NCHS), Centers for Disease Control and Prevention (CDC), and is the principal source of information on the health of the civilian noninstitutionalized household population of the United States.

The NHIS contains few of the variables in the format used by the modified ACR 2010 fibromyalgia criteria [1, 2]. Therefore, we developed a surrogate definition of fibromyalgia using similar, germane variables available in the 2012 NHIS. In the present paper we describe our efforts in testing and validating the surrogate definition in 415 rheumatology clinic patients who completed both an NHIS variables questionnaire and the 2010 modified criteria questionnaire. We then applied the surrogate definition to the 2012 NHIS data to describe the characteristics, predictors and outcomes for persons satisfying these criteria. In the results and discussion sections, when we use the term “fibromyalgia criteria” or fibromyalgia we are referring to the surrogate fibromyalgia definition, unless otherwise specified.

## Materials and Methods

### Subjects

The National Health Interview Survey (NHIS) is a multi-purpose health survey conducted by the National Center for Health Statistics (NCHS), Centers for Disease Control and Prevention (CDC). [3] The 2012 survey used a multi-stage clustered sample design, and over-sampled non-Hispanic black and Hispanic persons to allow for more accurate national estimates of health for these increasing minority populations. The survey contains four main modules: Household, Family, Sample Child, and Sample Adult. The first two modules collect health and sociodemographic information on each member of all families residing within a sampled household. Within each family, additional information is collected from one randomly selected adult (the “sample adult”) aged 18 years or older. The overall 2012 response rate was 79.7%.

Approximately one quarter of the NHIS adult sample were randomly selected to receive the Adult Functioning and Disability Supplement (AFD). In our analyses we merged the AFD dataset with other 2012 NHIS datasets according to NHIS instruction and weighting. The merged datasets assessed 8,446 individuals who represent a weighted population size of 225,726,257.

### Development of study variables and PSD scale

According to the ACR 2010 preliminary diagnostic criteria for fibromyalgia [2] and its modification for research purposes (research criteria), [1] a diagnosis of fibromyalgia can be made when levels of the Widespread Pain Index (WPI) and Symptom Severity Scale (SSS) are sufficiently high (WPI ≥ 7 and SSS ≥ 5, or WPI is 3–6 and SSS ≥ 9). The WPI is a 0–19 count of painful non-articular body regions and the SSS is a 0–12 measure of symptom severity that includes fatigue, sleep and cognitive problems. The polysymptomatic distress (PSD) scale represents a measure of the latent variable, polysymptomatic distress, and is calculated by taking the sum of WPI and SSS. Because of the definitional requirements of the fibromyalgia criteria, a positive case must have a PSD score of at least 12. However, not all subjects with a score ≥12 will satisfy fibromyalgia criteria. In the German general population study that used the research criteria, 38% of scores ≥12 were fibromyalgia criteria negative. [4] In that study a PSD score ≥13 minimized fibromyalgia misclassification. This was also found to be the case in the original research criteria study and follow-up studies. [1, 5] In general, failure to meet fibromyalgia criteria in PSD ≥12 is due to insufficiently high SSS. The NHIS captures joint pain and problems but not non-articular pain. Therefore, to develop surrogate measures, we first undertook a series of analyses using data from the National Data Bank for Rheumatic Diseases to determine if joints could substitute for non-articular regions in computing the WPI, the PSD scale and fibromyalgia diagnosis by modified ACR Research criteria. [1] We then performed a head-to-head comparison of NHIS surrogate measures with the modified ACR Research criteria in a prospective cohort of rheumatic disease patients.

### Validation of NHIS questions as surrogates for the modified ACR Research criteria

Using data from 14,396 patients with rheumatoid arthritis, osteoarthritis, or criteria positive fibromyalgia participating in survey research in the National Data Bank for Rheumatic Diseases (NDB), [6] we calculated the sum of self-reported tender joints (joint count), as well as the WPI, SSS and PSD scales. In addition we calculated a separate WPI (WPI-J) and a separate PSD score using joints (PSD-J) rather than non-articular regions. The Pearson correlation between PSD and PSD-J, WPI and the number of tender joints were 0.939, 0.947 and 0.860, respectively. Using the research criteria diagnosis of fibromyalgia as a Gold standard, the area under the Receiver Operating Curve (ROC) was 0.978 for PSD and 0.961 for PSD-J. With the widespread pain criterion (pain above and below the waist, on the left and right side of the body, and in the neck or back or thoracic spine) of the 1990 ACR criteria as a Gold Standard, [7] the ROC value was 0.964 for WPI and 0.919 for joint count.

### Head-to-head comparison of NHIS questions with 2010 modified ACR Research criteria

As the above results indicated that joints could be substituted for non-articular regions in fibromyalgia and polysymptomatic distress in the study setting, we undertook to further test these surrogate fibromyalgia and PSD variables in the clinical care setting. Two rheumatologists (RSK and MJB) administered a research questionnaire to consecutive patients in their rheumatology practices during ordinary clinical care. Participants were diagnosed with a variety of rheumatic diseases, including autoimmune diseases, osteoarthritis, localized pain disorders, and fibromyalgia. Side 1 of the questionnaire contained the research criteria questions and side 2 contained items taken directly from the NHIS questionnaire. The questionnaire is available as Appendix 1. Alternating patients completed side 1 or side 2 first. In the NHIS surveys, questions were generally read to participants by interviewers; however, interviewers were not possible in the clinical setting so we asked patients to read and answer the questions themselves from the questionnaire. Using the rheumatology clinic data, we first determined each patient’s PSD score and fibromyalgia criteria status from the Research Criteria questionnaire. The goal of the analysis of the clinical and NHIS data was to obtain a series of joint and symptom variables from the NHIS data that best predicted research criteria fibromyalgia, and then by logistic regression and ROC analysis to determine the classification and discrimination ability of these variables. Following that, we would regress the research criteria PSD score on the predictor variables. The intercept and predictor variable coefficients from this model would subsequently be used to calculate a PSD score in the NHIS data sets. NHIS variable questions and variables differed from research criteria variables with respect to wording, content, scoring and context, so simple 1:1 variable correspondence was not possible.

There were 415 patients who completed the clinic questionnaire. The average missing items per questionnaire was 1.4, with percent missing for items ranging between 0 and 19.2%. 274 questionnaires were completed without missing data. We used multiple imputation by chained equations [8] and 10 iterations to develop a complete imputed set of 415 questionnaires, and used the imputed data to develop PSD predictors for use in the main NHIS data sets. Although results of the imputed and non-imputed data sets were similar, use of the imputed data allowed use of the full sample.

As all NHIS variables on the rheumatologist administered questionnaire were candidates for model inclusion, explored a number of regression models to obtain best predictors combined with clinical judgment that respected the intent of the research criteria. For the estimated PSD scores, we considered 3 models: 1) a linear regression model that combined joints and symptoms, 2) a similar model that separately analyzed joints and symptoms, and 3) a linear regression model that combined joints into regions and regressed on the regions and symptoms rather than on individual joints and symptoms. Although all models yielded similar results, model 1 was the simplest to use and we selected that as the PSD to be used in analysis of NHIS data.

The NHIS definition found to best approximate 2010 ACR criteria included specific multiple painful joint sites (right and left hand/wrist, elbow, shoulder, hip and knee, and unpaired sites of low back pain, face pain and abdominal pain. Tiredness (fatigue) and concentration ability were the included symptom variables.

The R-Squared for measured and predicted PSD was 0.781. Collinearity diagnostics on non-imputed data showed no evidence of elevated collinearity scores (variance Inflation factor (VIF) = 1.83). Using a logistic model, we tested NHIS predictors against observed research criteria positivity. The area under the receiver-operating curve (AUC ROC) was .946. 88.1% of cases were properly classified, and the sensitivity/specificity was 74.7%/93.4%. Ten-fold cross validation with 100 replications showed a ROC of .901 (95% CI .897, .902).

### Determination of optimum case definition for fibromyalgia using NHIS questions

Based on study analyses, the Youden index, [9] and prior data, [1, 4] we selected a PSD score ≥13 to designate a fibromyalgia case because study variables did not permit a computationally valid measure of SSS. At this level, 85.5% with a PSD ≥13 satisfied the ACR 1990 criteria definition of widespread pain. [7] In the recent German general population survey of 2,445 subjects, 82.7% of fibromyalgia positive participants had widespread pain. [4]

### Independent variables

The other variables and definitions used in this study are included in Appendix 2. The detailed description of the variables can be found in the NHIS publications. [3] The definition for obesity comes from the World Health Organization: Underweight (BMI 18.5 kg/m2), Normal weight (BMI 18.5–24.9 kg/m2), "Overweight (BMI 25.0–29.9 kg/m2)", "Obese (BMI >30.0 kg/m2)" [10]

### Statistical methods

We used publicly available NHIS data for 2012. [3] Analyses were performed using Stata 13.1 survey statistical methods that incorporated NHIS supplied probability weights, strata and primary sampling unit data. [11] The primary methods of analysis included logistic, linear, Poisson and interval censored regression for binary, continuous, count and interval categorical data, respectively. Independent variables were modeled to include interaction effects, followed by the determination of marginal probabilities (or means), from which covariate adjusted odds ratios, coefficients and incident rate ratios were determined.

### Ethics Statement

All authors declare they have no competing interests. The study was approved by the Via Christi institutional review board, Wichita, KS.

## Results

### Prevalence

In the US population of persons ≥18 years of age in 2012, 1.75% (95% CI 1.42, 2.07), or 3.94 million, satisfied study specific criteria for fibromyalgia (Table 1). The prevalence was lowest in the 18–29 age group (0.76% (0.05, 1.46)) and rose to 2.41% (1.49, 3.33) in the 50–59 years age group, from which there was no significant difference in prevalence compared with older age groups. Among those with fibromyalgia, 83.57% satisfied the ACR 1990 criterion for widespread pain compared to 5.21% without fibromyalgia. The mean NHIS PSD score was 2.75 (2.66, 2.85) overall, and was 16.10 (15.47, 16.74) and 2.04 (1.97, 2.12) in those with and without fibromyalgia, respectively. The mean NHIS PSD score for the 27% of fibromyalgia criteria positive subjects who had a physician’s diagnosis of fibromyalgia was 16.8 (15.86, 17.74) compared with 15.21 (14.43, 16.00) for the 73% without a physician’s diagnosis (p = 0.014).

**Table 1:** Prevalence of fibromyalgia in adult general population (2012).

*Age group*	*Population N (Weighted)* *	*FM Total N (Weighted)*	*FM Total (%)*	*FM Women (%)*	*FM Men (%)*
18–29	48577237	367129	0.76 (0.05, 1.46)	0.82 (0.13, 1.52)	0.69 (-0.53, 1.91)
30–39	37933978	532162	1.40 (0.65, 2.16)	2.35 (0.96, 3.75)	0.41 (-0.10, 0.92)
40–49	41478955	943235	2.27 (1.32, 3.23)	3.24 (1.56, 4.91)	1.26 (0.29, 2.23)
50–59	40150510	967111	2.41 (1.49, 3.33)	2.52 (1.27, 3.78)	2.29 (0.99, 3.60)
60–69	31271847	641512	2.05 (1.15, 2.95)	3.45 (1.78, 5.13)	0.48 (0.05, 0.92)
70–79	16412943	331547	2.02 (0.82, 3.22)	2.61 (0.76, 4.46)	1.34 (0.00, 2.76)
80–85	9900787	159629	1.61 (0.50, 2.72)	2.10 (0.46, 3.74)	0.76 0.00, 1.85)
Total	225726257	3942325	1.75 (1.42, 2.07)	2.38 (1.85, 2.92)	1.06 (0.71, 1.41)

FM = fibromyalgia.

* Based on a study population of 8446.

Based on subject self-report, the NHIS separately categorized physician reported “arthritis” [“Have you EVER been told by a doctor or other health professional that you have some form of arthritis, rheumatoid arthritis, gout, lupus, or fibromyalgia (fy-bro-my-AL-jee-uh)?”] and back pain diagnoses so that the two categories were separate. Among persons satisfying NHIS fibromyalgia criteria, 27.0% had a physician’s diagnosis of fibromyalgia, 15.3% of rheumatoid arthritis, 3.3% of gout, 1.4% of lupus and 21.7% of low back pain. After excluding overlaps with physician-diagnosed fibromyalgia, 47.5% had a diagnosis of “arthritis.” The NHIS case-finding question for arthritis appears to be valid for surveillance purposes. [12]

### Demographic and behavioral variables

**Fig 1:**
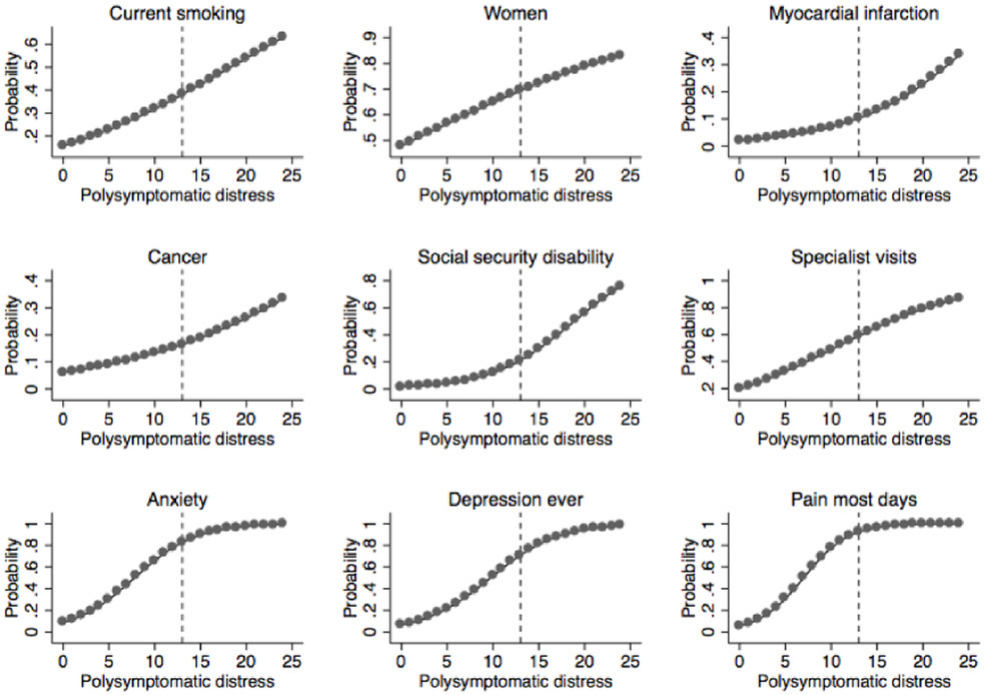
Predicted probabilities (unadjusted) of selected variables in the 2012 National Health Interview Survey as a function of polysymptomatic distress scores.

The prevalence of fibromyalgia was 2.38% (1.85, 2.92) in women compared with 1.06% (0.71, 1.41) in men, odds ratio (OR) 2.28 (1.52, 3.42), P <0.001 (Table 2). With respect to ethnicity, there was no significant difference among Hispanics, non-Hispanic whites and non-Hispanic blacks compared with non-Hispanic Whites, but Asians had a significantly lower fibromyalgia prevalence, 0.20% (-0.01, 0.04) and “all other races” (more than one race and/or American Indian/Alaska Native (AIAN), Asian, and Native Hawaiian and Other Pacific Islander) a significantly greater prevalence, 7.35% (-1.03, 15.74). Fibromyalgia was more common in US citizens and in persons living in the Midwest. Other predictors of fibromyalgia included being divorced or separated, being obese or a current smoker, and not having a college education. The effect of PSD as a continuous variable on the probability being a woman or a current smoker is shown in Fig 1.

**Table 2:** Demographic characteristics of persons with and without fibromyalgia in the National Health Interview Survey (2012).

*FM Predictors*	*Percent or mean* * *in Population/ Percent or Mean (SE)* * *in FM*	*Condition Present*: *FM% or Mean (SE)* * *in FM*	*Condition Absent*: *FM % or (Mean SE)* * *In FM*	*Odds Ratio Coefficient IRR* † *(95% CI)*	*P-value*
All Participants (FM% = 1.75)					
Age (years): Mean (SE)	46.6 (0.26)*	51.4 (1.57)*	46.5 (0.26)*	4.84 (1.76, 7.93)‡	0.002
Sex (% female)	51.7/70.7	2.39	1.06	2.28 (1.52, 3.42)	0.000
Ethnicity/Race					
Non-Hispanic White (base) %	67.2/73.5	1.91		1	
Hispanic %	15.1/10.5	1.35	1.22	0.72 (0.43, 1.24)	0.241
Non-Hispanic Black %	11.8/12.7	1.90	1.87	1.02 (0.59, 1.74)	0.942
Non-Hispanic Asian %	5.3/0.6	0.20	0.20	0.11 (0.03, 0.46)	0.003
Non-Hispanic other races %	0.7/2.7	7.35	6.85	3.95 (1.27, 12.34)	0.018
US Citizen: Yes v. No %	91.3/97.4	1.83	0.53	3.44 (1.44, 8.25)	0.006
Midwest v. N.E, South, West %	23.4/36.9	2.72	1.44	1.88 (1.26, 2.82)	0.002
Marital status					
Married/cohabiting (base) %	60.7/51.3	13.7		1	
Never married %	22.2/17.5	2.24	13.7	1.63 (0.83, 3.23)	0.159
Divorce/separated %	11.1/22.9	2.84	13.7	2.07 (1.38, 3.11)	0.000
Widowed %	5.9/8.3	2.02	13.7	1.47 (0.75, 2.8)	0.262
Not college graduate %	72.9/87.7	2.17	0.74	2.91 (1.72, 4.95)	0.000
Obese v. Non-obese %	30.1/46.8	2.53	1.37	1.85 (1.25, 2.72)	0.002
Current smoker %	19.0/38.5	3.67	1.31	2.79 (1.91, 4,07)	0.000
Alcohol: lifetime abstainers %	35.9/41.4	1.94	1.63	1.19 (0.82, 1.74)	0.356
Alcohol: (days per yr) mean SE	5.4 (4.9)*	4.2 (2.8)*	5.4 (0.50)*	0.78 (0.38, 1.62) †	0.463

Age and sex are unadjusted, Ethnicity/race are adjusted for age and sex; all other variables are adjusted for age, sex and ethnicity/race

* Mean (SE)

† Incident rate ratio

‡OLS regression coefficient

FM = fibromyalgia.

### Comorbid conditions

Table 3 shows the relation between fibromyalgia status and lifetime comorbid conditions. Column 2 contains the percent with the disorders in the overall population compared with persons who satisfy study fibromyalgia criteria. For example, 8.3% of the overall population has diabetes while 23.3% with fibromyalgia has diabetes. Columns 3 and 4 display fibromyalgia prevalence in those with and without the comorbid condition. Column 5 provides the odds ratio and 95% confidence for the condition as a function of fibromyalgia status. All medical disorders are more common in persons with fibromyalgia. Consistently higher odds ratios are associated with mental disorders. 15.3% of fibromyalgia positive participants reported having rheumatoid arthritis, confirming the increase in rheumatoid arthritis seen in clinical practice. The association of cancers with fibromyalgia was OR 1.77 (0.99, 3.17) p = 0.053. A validation variable is available for diabetes self-report: compared to those without fibromyalgia, the odds ratio for anti-diabetic medication use in fibromyalgia is 2.28 (1.39, 3.77), p = 0.001. Finally, the relation of PSD and the probability of lifetime cancer or myocardial infarction in shown in Fig 1.

**Table 3:** Comorbid medical and psychiatric illness association with Fibromyalgia.

*Comorbid Condition (Ever unless described)*	*Percent in Population / Percent in FM*	*Condition Present FM Prevalence %* *	*Condition Absent (FM Prevalence %)* *	*Odds Ratio (95% CI)* *	*P-value* *
All Persons (Baseline)			1.75		
Myocardial infarction	2.9/8.6	4.47	1.65	2.88 (1.31, 3.64)	0.009
Heart disease	6.6/15.9	3.73	1.59	2.47 (1.43, 4.28)	0.001
Stroke	2.4/7.7	3.79	1.67	2.37 (1.09, 5.15)	0.029
Liver disease	1.1/6.1	7.43	1.66	5.07 (2.11, 12.21)	0.000
Kidney (weak or failing)	1.6/6.8	6.23	1.66	4.16 (1.97, 8.81)	0.000
Hypertension	29.7/54.2	2.74	1.22	2.32 (1.44, 3.75)	0.001
Diabetes	8.4/23.3	3.88	1.50	2.73 (1.74, 4.30)	0.000
Emphysema	1.7/5.5	3.05	1.70	1.85 (0.84, 4.04)	0.125
COPD	2.8/12.0	4.01	1.62	2.64 (1.47, 4.75)	0.001
Asthma	11.7/29.6	3.88	1.42	2.88 (1.88, 4/04)	0.000
Stomach ulcer	6.46/263	5.38	1.40	4.15 (2.59, 6.66)	0.000
Rheumatoid arthritis	2.3/15.3	7.72	1.53	5.73 (3.25, 10.12)	0.000
Lupus	0.4/1.4	2.80	1.74	1.65 (0.46, 5.86)	0.436
Migraines in 0–3 months	14.2/56.2	6.26	0.90	7.68 (4.70, 12.53)	0.000
Hepatitis	2.6/3.9	1.99	1.73	1.15 (0.39, 3.37)	0.795
Influenza/pneumonia	23.5/54.0	3.53	1.10	3.38 (2.26, 5.07)	0.000
Depression	14.2/62.7	5.84	0.81	7.9 (4.94, 12.65)	0.000
Phobias	5.3/32.9	8.32	1.26	7.62 (4.84, 11.99)	0.000
Bipolar illness	2.5/17.1	9.01	1.50	7.03 (3.21, 15.39)	0.000
Mental health (other)	4.0/26.6	10.35	1.34	9.40 (5.39, 16.41)	0.000
All Cancers	7.6/15.0	2.82	1.63	1.77 (0.99, 3.17)	0.053
Ca Bone	0.07/0.7	11.12	1.74	7.88 (0.74, 83.58)	0.086
Ca Breast	1.2/0.7	0.07	1.76	0.37 (0.11, 1.23)	0.105
Ca Colon	0.4/1.1	5.09	1.73	3.16 (0.49, 20.34)	0.224
Ca Liver	0.02/0.5	46.34	1.73	41.25 (2.99, 569.57)	0.006
Ca Pancreas	0.03/0.4	30.26	1.74	32.86 (2.69, 401.53)	0.006
Ca Prostate	0.8/0.9	3.24	1.74	1.93 (0.69, 5.35)	0.205

*Adjusted for age, sex, ethnicity, education, obesity, smoking, US region.

Ca = Cancer. COPD = Chronic obstructive lung disease.

### Work and disability

As shown in Table 4, 55.8% of persons with fibromyalgia <65 years of age reported that they were unable to work now because of health compared with 5.8% without fibromyalgia (p <0.001). Of persons with fibromyalgia,71.3% of men and 41.3% of women reported that they currently could not work because of health (p = 0.021). Of persons with fibromyalgia, 50.5% of persons <65 years of age filed Social Security disability applications at any time compared with 5.8% without fibromyalgia (p<0.001). Among those with fibromyalgia, 65.7% of men and 36.3% of women filed applications (p = 0.026). Disability payments were made in the last year to 30.2% of those with fibromyalgia compared with 2.8% without fibromyalgia (p <0.001). 28.1% of women and 32.4% of men with fibromyalgia received Social Security disability payments in the last year (p = 0.674). The effect of PSD on the probability of receiving Social Security disability payments in the last year is shown in Fig 1.

**Table 4:** Work disability and inpatient and outpatient utilization in persons with and without fibromyalgia in the 2012 National Health Interview Survey.

*Logistic regression*	*FM+*	*FM-*	*OR (95% CI)*	*P-value*	*FM+ (male)*	*FM+ (female)*	*P-value M. v. F.*
SS disability Application ever (%)	50.5	5.8	8.68 (6.37, 11.81)	0.000	65.7	36.3	0.026
SS disability payments Last year (%)	30.2	2.8	10.91 (7.58, 15.70)	0.000	32.4	28.1	0.674
Unable to work now because of health (%)	55.8	5.8	9.62 (7.35, 12.61)	0.000	71.3	41.3	0.021
Hospitalized in year (%)	17.7	8.8	2.03 (1.42, 2.89)	0.000	19.9	15.9	0.549
Multiply hospitalized (%)	32.0	25.0	1.32 (0.78, 2.23)	0.308	39.4	26.4	0.470
Specialist visit in year (%)	49.7	25.2	1.97 (1.64, 2.37)	0.000	45.1	54.0	0.348
Generalist visit in year (%)	72.6	66.3	1.09 (0.98, 1.22)	0.102	64.9	78.0	0.567
Problem paying medical bills (%)	37.5	17.4	2.15 (1.53, 3.04)	0.000	36.3	38.7	0.842
Linear regression	*FM+*	*FM-*	*Marginal effect (95% CI)*	*P-value*	*FM+ (male)*	*FM+ (female)*	*P-value M*. *v*. *F*.
	Mean (S.E)				Mean (S.E)		
Annual medical office visits Mean (S.E.)	8.69 (0.70)	3.84 (0.07)	4.85 (3.47, 6,24)	0.000	8.29 (1.19)	9.06 (0.74)	0.579

Adjusted for age, sex, ethnicity/race. Work and disability data restricted to person 65 years of age.

The NHIS also provides some measures of utilization of medical services. Hospitalization was experienced by 17.7% of those with fibromyalgia compared with 8.8% without fibromyalgia. Among those hospitalized in a one-year period, multiple hospitalizations were not more common in fibromyalgia (32.0%) than in those without fibromyalgia (25.0%). The probability of seeing a medical specialist in the last year was increased in those satisfying fibromyalgia criteria (49.7% vs. 25.2%, p <0.001), but the probability of general medical visits was not increased (72.6% vs. 66.3%, p = 0.102). The overall 1-year number of medical visits for the two groups was 8.69 vs. 3.88, p <0.001, or 4.85 (3.47, 6.2) more medical office visits. Out of pocket medical expenses were not increased for those with fibromyalgia (OR 0.86 (0.55, 1.34, p = 0.509) by ordered logistic regression. PSD levels were associated with the probability of specialist visits in Fig 1.

### Psychological symptoms and functional ability

All measures of psychological status, fibromyalgia symptoms (insomnia, memory loss and fatigue), pain and functional status were substantially increased or more common in fibromyalgia (Table 5). More than 43% of those with fibromyalgia used anxiety and depression medications, and pain was reported to occur on most or all days by 87%. Compared to those without fibromyalgia, odds ratios >13 were noted for much difficulty or unable to climb stairs and much difficulty or unable to do self-care. The strong association between PSD and anxiety, depression and pain is shown in Fig 1.

**Table 5:** Symptom and functional ability variable association with Fibromyalgia.

*Symptom variables*	*Percent in Population / Percent in FM*	*Condition Present FM Prevalence %* *	*Condition Absent (FM Prevalence %)* *	*Odds Ratio (95% CI)* *	*P-value* *
All Participants (FM% = 1.75)					
Often anxious (year) %	18.9/64.8	5.67	0.77	7.88 (4.92, 12.64)	0.000
Uses anxiety meds %	9.2/43.6	7.40	1.10	7.33 (4.91, 10.96)	0.000
Uses depression meds %	8.7/44.7	7.80	1.07	7.93 (5.26, 11.94)	0.000
Insomnia past year %	19.5/67.5	5.55	0.72	8.07 (4.96, 13.14)	0.000
Memory loss past year %	4.7/43.6	15.57	1.03	18.22 (10.46, 31.74)	0.000
Fatigue more than 3 days last year %	14.6/81.3	8.82	0.03	25.02 (13.55, 46.14)	0.000
Pain: most or all days %	17.3/86.6	8.41	0.29	32.55 (15.79, 67.13)	0.000
Climbing stairs: much difficulty or unable %	4.8/39.2	14.41	1.11	15.45 (7.96, 29.98)	0.000
Walking ¼ mile: much difficulty or unable %	1.26/6.84	8.02	1.65	5.30 (2.38, 11.81)	0.000
Self-care: much difficulty or unable %	0.7/7.5	17.34	1.63	13.60 (6.36, 29.08)	0.000

*Adjusted for age, sex, and ethnicity

## Discussion

The picture of criteria positive fibromyalgia that comes from National Health Interview Survey data is one of high levels of self-reported pain, non-pain symptoms, comorbidity and psychological distress. It includes substantial medical costs and high rates of Social Security disability and work disability; and it is associated with gender, education, ethnicity, citizenship and unhealthy behaviors. These data are important because they verify in a population-based unbiased source many, but not all, observations about fibromyalgia made in the clinic. [13–19]

Fibromyalgia is the name given to persons with high levels of characteristic symptoms, [20]particularly those related to pain. The dividing points between fibromyalgia and not fibromyalgia in the ACR 1990 and 2010 criteria were based on a criteria committee’s evaluation of symptom severity. With a prevalence of 1.75% in the current study and 2.1% in the large German population study, [4] fibromyalgia stands at the 98^th^ percentile of the spectrum of polysymptomatic distress[21], with a PSD diagnostic cut point of 12–13 that yields the most accurate classification of the syndrome. While the results shown in Tables 2–5 demonstrate a clear and substantial separation between fibromyalgia criteria positive and negative cases, Fig 1 shows that separation into two groups is artificial; persons with mild symptoms flow into those across the 12–13 boundary.

To have clinical fibromyalgia, for which one receives specific treatment and an insurance diagnosis, requires a diagnosis by a physician. [20, 22]Most persons we identified as meeting our study’s fibromyalgia criteria did not report a physician’s diagnosis of fibromyalgia. Instead, 15.3% had a diagnosis of rheumatoid arthritis, 3.3% of gout, 1.4% of lupus, 21.7% of low back pain, and excluding overlaps with physician diagnosed fibromyalgia, 47.5% had a diagnosis of “arthritis” that was not further defined in the NHIS data. This may reflect a clinical tendency to preferentially attribute symptoms to other clinical disorders rather than invoking a second diagnosis of fibromyalgia. It also seems to suggest that fibromyalgia symptoms are not restricted to otherwise healthy persons but also commonly occur in the setting of concomitant medical disease, such as “fibromyalgic rheumatoid arthritis”.[23] It seems clear that differences in a clinician’s interpretation of symptoms will have substantial impact on the nature of treatments offered and covered by private and governmental health care insurance plans. The reasons for survey participants not reporting a clinical diagnosis of fibromyalgia in the face of substantial fibromyalgia symptoms cannot be discerned from our data. Possible causes for non-diagnosis include the presence of other medical diagnoses, lack of knowledge; or a disagreement about the nature and meaning of symptoms, and how they should be characterized. [24–32]Among the specific important findings of this study is the association of our surrogate fibromyalgia diagnosis with demographic characteristics (Table 1). Fibromyalgia is more likely to be found in women, although of a substantially smaller magnitude than observed in clinical cohorts. Unlike what is seen in clinically-derived cohorts, fibromyalgia was equally experienced across ethnicities, with the exception of fibromyalgia being less frequent in Asians. As expected, being divorced, obese or a smoker is more likely in those with fibromyalgia, while college level education has a protective effect. These factors reinforce the importance of social disadvantage on the risk of fibromyalgia and polysymptomatic distress. [33–35] The finding that citizenship is associated with a 3-fold increases in the odds of fibromyalgia suggests a strong predisposing role for individuals fully acculturated into the general population, and offers support to the idea that symptoms may be understood and manifested differently by different cultures. [36–38]

The association of lifetime comorbid illnesses with our surrogate fibromyalgia diagnosis is a central finding of this study (Table 3). People with fibromyalgia had marked increases in major medical conditions. For example, myocardial infarction, hypertension and diabetes occurred more than twice as commonly in those with fibromyalgia. We also noted that fibromyalgia occurs more frequently in rheumatoid arthritis and lupus. We found that fibromyalgia also may be more common in some cancers, although our estimates are unstable and uncertain, probably relating to small sample sizes in those with cancer; and we verified increased rates of depression and other mental illnesses. Similar physical and mental illness associations have been reported in a longitudinal database of rheumatoid arthritis patients who satisfied fibromyalgia criteria, [33] in a clinical practice setting, [39] and in diagnosed fibromyalgia patients in a health insurance database. [13] Investigations of antecedent factors in the development of widespread pain have implicated childhood trauma and psychological abnormalities. [40, 41] It seems possible that the comorbid physical illnesses that we identified may represent antecedent stressors. Alternatively, they might in part represent mediated illnesses related to existing behavioral factors, such as obesity and smoking history, as well as to the presence of chronic pain. Regardless, this observation serves to remind clinicians that human disease often comes with symptoms that cannot be easily attributed to the disease itself. Awareness of the strong relationship between comorbidity and fibromyalgia symptoms may aid clinicians in reducing unnecessary medical testing and patients’ health concerns.

The contemporary intellectual ferment in fibromyalgia research has involved the increasing knowledge of neurobiologic mechanisms of pain and symptoms. [42] Our data, which suggests importance of psychosocial data and social construction mechanistics, is not adverse to the contemporary biomedical model, as the typology of illness has three superordinate categories: biological, psychological, and environmental or socio-cultural; and risk factors are distributed across these categories [43, 44] However, at the current time fibromyalgia diagnosis is almost completely dependent on symptoms. [45–48] Because there is no generally accepted external Gold standard for diagnosis, symptoms as polysymptomatic distress are diagnosis. But, there is no clear distinguishing point where fibromyalgia stops being fibromyalgia and becomes some other illness or no illness at all (Fig 1). Values surrounding PSD values of 12–13 do not truly distinguish different levels of symptoms. If fibromyalgia symptoms of diminishing severity are found all the way down to zero PSD, then “central nervous system origins of or amplification of pain” [42] may not be a disease process, but the normal way that humans respond to certain physiological and mental stresses (Fig 1). [4] Data are accumulating that polysymptomatic distress is a marker and contributing factor to illness at levels below that found in fibromyalgia. [49, 50]

In addition, as noted above, fibromyalgia in the community is an optional diagnosis for many physicians. In the current report, subjects meeting NHIS fibromyalgia criteria report receiving diagnoses other than fibromyalgia. Additionally, as we report in a companion NHIS paper (in submission) [51] about 75% of persons reporting a diagnosis of fibromyalgia by a physician or health profession fail to meet surrogate fibromyalgia criteria. It seems possible that strong cultural pressure, including direct-to-patient advertising drives toward diagnosis. [52–55]

**[18, 56–58]**This study has a number of important limitations. As noted above, questions from the NHIS questionnaire differed in many ways from the ACR-based research questionnaires so a 1:1 correspondence between variables was not possible. While the research criteria inquired about broad predominantly non-articular regions, the NHIS questionnaire directed attentions to specific joint regions. Symptom variables also differed in their wording, severity and timing, and there were fewer symptom variables available in the NHIS data than used by the research criteria. The ACR criteria do not exclude persons because of other illnesses, including painful illnesses. In a previous study, we wrote “We also made no exclusions for the presence of ‘another disorder that would otherwise sufficiently explain the pain.’ This is a controversial requirement because it is not certain how to define such disorders. Even so, our epidemiology studies, as with most fibromyalgia epidemiology studies, did not have sufficient data to make such exclusions.”**[4]** The surrogate PSD score that we used in the NHIS analyses had a shorter range than the original PSD, perhaps related to these overall differences. Also, questions about pain location were only asked to persons reporting pain disorders and rheumatic diseases, which artificially decreases the percentage of widespread pain reported in the entire NHIS population.

The most important concern is how well our PSD cut point would have agreed with the unmeasured but actual cut point had we been able to administer the research fibromyalgia questionnaire to the NHIS participants. The best evidence in support of our data comes from comparison with the 2012 German population study that used the actual fibromyalgia research questionnaire. With regard to the following: length of the PSD scale US 0–24, Germany 0–27; mean PSD US 2.28, Germany 3.0; fibromyalgia cut point US 13, Germany 13; fibromyalgia %positive US 1.75, Germany 2.1; age US 46.6, Germany 50.2; female US 51.7% Germany 53.5%; widespread pain when fibromyalgia positive US 83.6 Germany 82.7%. The results are close between the countries. If we assume that an NHIS cut point should have been 12 instead of 13 used in this study, the fibromyalgia prevalence would have been 2.26 instead of 1.75. Odds ratio differences would be slight. Overall, we believe the PSD scale and fibromyalgia estimate have sufficient accuracy for the analyses described.

Despite these limitations, these data have implications on clinical practice and service delivery. Fibromyalgia is better conceptualized as a symptom continuum that is influenced by physical and psychological stressors rather than a discrete diagnosis. It is important for clinicians to acknowledge their patients’ polysymptomatic distress yet take care to avoid over-attribution of those symptoms to specific medical conditions, which potentially can lead to unnecessary medical testing and over-treatment. The data also remind that fibromyalgia diagnosis represents a very high degree of symptom severity that is only seen in 1.75% of the US population based on our surrogate measure. Our finding that 75% of survey participants reporting a clinical diagnosed fibromyalgia do not meet study specific severity criteria suggests that clinicians should be careful not to over-interpret reports mild and moderate amounts of polysymptomatic distress into fibromyalgia. The use of descriptive polysymptomatic distress categories, such as mild, moderate, and severe symptoms, rather than using a discreet dichotomous definition for fibromyalgia may represent a useful alternative manner in which to consider fibromyalgia symptoms.[59]

In summary, we have developed study specific surrogate criteria that allowed us to study fibromyalgia and polysymptomatic distress in the US National Health Interview Survey, the principal source of information on the health of the civilian noninstitutionalized household population of the United States. Based on current symptom severity standards for determining fibromyalgia, we found an approximate calculated prevalence of 1.75%. However, we also observed that fibromyalgia symptoms, as measured by the polysymptomatic distress scale, were continuous, and that there was no clearly defined cut-point that separated fibromyalgia from non-fibromyalgia. Persons with a fibromyalgia diagnosis had high levels of self-reported pain, non-pain symptoms, comorbidity and psychological distress; substantial medical costs and high rates of Social Security disability and work disability. Fibromyalgia diagnosis was associated with gender, education, ethnicity, citizenship and unhealthy behaviors. Although the direction of causality is often unclear in cross-sectional data, the physical illness comorbidities of Table 3 suggest that stress related to physical illness lies on the causal pathway to fibromyalgia development. Psychological status may also play a role, as other research points to some degree of causality. [18, 56–58]. Finally, the “under diagnosis” of clinical fibromyalgia and the relation to demographic factors to diagnosis points toward the possible influence of social and cultural factors on diagnosis acceptability.

## Supporting Information

S1 File Questionnaire Administered to in Rheumatology Offices. (PDF)

S2 File Independent variables evaluated in NHIS fibromyalgia study. (DOCX)
