# Supplementary material for: The Prevalence and Characteristics of Fibromyalgia in the 2012 National Health Interview Survey
Source: PLoS One. 2015 Sep 17;10(9):e0138024. doi: 10.1371/journal.pone.0138024 (PMC4575027; doi:10.1371/journal.pone.0138024)
Supplement: S1 File — (PDF) [file pone.0138024.s001.pdf]

☐ Start on this side first

I. Using the following scale, indicate for each item the level of severity **over the past week** by checking the appropriate box.

0: No problem
1: Slight or mild problems; generally mild or intermittent
2: Moderate; considerable problems; often present and/or at a moderate level
3: Severe; continuous, life-disturbing problems

| | | | | |
|---|---|---|---|---|
| Fatigue | ☐ 0 | ☐ 1 | ☐ 2 | ☐ 3 |
| Trouble thinking or remembering | ☐ 0 | ☐ 1 | ☐ 2 | ☐ 3 |
| Waking up tired (unrefreshed) | ☐ 0 | ☐ 1 | ☐ 2 | ☐ 3 |

II. During the **past 6 months** have you been bothered by any of the following symptoms?

| | | |
|---|---|---|
| Pain or cramps in lower abdomen | ☐ Yes | ☐ No |
| Depression | ☐ Yes | ☐ No |
| Headache | ☐ Yes | ☐ No |

III. Please indicate below if you have had **pain or tenderness over the past 7 days** in each of the areas listed below.
Please make an X in the box if you have had pain or tenderness. Be sure to mark both right side and left side separately.

| | | |
|---|---|---|
| ☐ Shoulder, Lt.<br>☐ Shoulder, Rt. | ☐ Upper Leg, Lt.<br>☐ Upper Leg, Rt. | ☐ Lower Back<br>☐ Upper Back<br>☐ Neck |
| ☐ Hip, Lt.<br>☐ Hip, Rt. | ☐ Lower Leg, Lt.<br>☐ Lower Leg, Rt. | |
| ☐ Upper Arm, Lt.<br>☐ Upper Arm, Rt. | ☐ Jaw, Lt.<br>☐ Jaw, Rt. | ☐ No pain in any of these areas |
| ☐ Lower Arm, Lt.<br>☐ Lower Arm, Rt. | ☐ Chest<br>☐ Abdomen | |

IV. Overall, were the symptoms listed in I - III above generally present for at **least 3 months**? ☐ Yes ☐ No

Draft

☐ Start on this side first

DURING THE PAST 30 DAYS, have you had any symptoms of pain, aching, or stiffness in or around a joint? ☐ Yes ☐ No

Which joints are affected?

| | | |
|---|---|---|
| ☐ Shoulder, Lt.<br>☐ Shoulder, Rt. | ☐ Knee, Lt.<br>☐ Knee, Rt. | ☐ Elbow, Rt.<br>☐ Elbow, Lt. |
| ☐ Hip, Lt.<br>☐ Hip, Rt. | ☐ Fingers/thumb, Lt.<br>☐ Finger/thumb, Rt. | ☐ Toes, Lt.<br>☐ Toes, Rt. |
| ☐ Wrist, Lt.<br>☐ Wrist, Rt. | ☐ Ankle, Lt.<br>☐ Ankle, Rt. | |

The following questions are about pain you may have experienced in the PAST THREE MONTHS.
Please refer to pain that LASTED A WHOLE DAY OR MORE. Do not report aches and pains that are fleeting or minor.

Neck pain? ☐ Yes ☐ No

Low back pain? ☐ Yes ☐ No

Did this pain spread down either leg to areas below the knees? ☐ Yes ☐ No

Facial ache or pain in the jaw muscles or the joint in front of the ear? ☐ Yes ☐ No

DURING THE PAST 12 MONTHS:

Did you have abdominal pain? ☐ Yes ☐ No

Have you had regularly had insomnia or trouble sleeping? ☐ Yes ☐ No

Did you have severe headache or migraine? ☐ Yes ☐ No

Do you have difficulty remembering or concentrating?

☐ No difficulty ☐ Some difficulty ☐ A lot of difficulty ☐ Cannot do at all/unable to do

Have you been depressed? ☐ Yes ☐ No

Thinking about being very tired or exhausted in the last 3 months, how would you describe the level of tiredness? Would you say: ☐ None ☐ A little ☐ A lot ☐ Somewhere in between a little and a lot

Have you EVER been told by a doctor or other health professional that you had depression? ☐ Yes ☐ No
