# Supplementary material for: The Prevalence and Characteristics of Fibromyalgia in the 2012 National Health Interview Survey
Source: PLoS One. 2015 Sep 17;10(9):e0138024. doi: 10.1371/journal.pone.0138024 (PMC4575027; doi:10.1371/journal.pone.0138024)
Supplement: S2 File — (DOCX) [file pone.0138024.s002.docx]

Appendix II. Independent variables evaluated in NHIS fibromyalgia study

| Table Label (if used in table) | Name | National Health Interview Survey label | Coding in study |
| --- | --- | --- | --- |
| Specialist visit in year (%) | ahcsyr8 | AAU.211_01.000: Seen/talked to a medical specialist, past 12 m | Yes/no |
| Generalist visit in year (%) | ahcsyr9 | AAU.211_02.000: Seen/talked to a general doctor, past 12 m | Yes/no |
| Annual medical office visits Mean (S.E.) | ahcnoyr2 | AAU.280_00.000: Total number of office visits, past 12 m | Number |
|  | shtflu2 | AAU.310_00.000: Flu shot past 12 m | Yes/no |
| Hepatitis | ahep | AAU.350_00.000: Ever had hepatitis | Yes/no |
| Liver disease | livev | AAU.450_00.010: Ever told you had any kind of chronic/long-term liver condition | Yes/no |
| Hypertension | hypev | ACN.010_00.000: Ever been told you have hypertension | Yes/no |
| Myocardial infarction | miev | ACN.031_03.000: Ever been told you had a heart attack | Yes/no |
| Heart disease | hrtev | ACN.031_04.000: Ever been told you had a heart condition/disease | Yes/no |
| Stroke | strev | ACN.031_05.000: Ever been told you had a stroke | Yes/no |
| Emphysema | ephev | ACN.031_06.000: Ever been told you had emphysema | Yes/no |
| COPD | copdev | ACN.035_00.000: Ever been told you had COPD | Yes/no |
| Asthma | aasmev | ACN.080_00.000: Ever been told you had asthma | Yes/no |
| Ulcer | ulcev | ACN.110_00.000: Ever been told you have an ulcer | Yes/no |
| Influenza/pneumonia | aflupnev | ACN.121_00.030: Ever told you had influenza/pneumonia | Yes/no |
| Phobias | phobiaev | ACN.121_00.110: Ever told you had phobia or fears | Yes/no |
| Bipolar illness | bipdis | ACN.121_00.140: Ever told you had Bipolar Disorder | Yes/no |
| Depression | adeprsev | ACN.121_00.150: Ever told you had depression | Yes/no |
| Mental health (other) | mhdothev | ACN.121_00.170: Ever told you had other mental health disorders | Yes/no |
|  | memlosyr | ACN.125_00.120: Had memory loss, past 12 months | Yes/no |
|  | aabdomyr | ACN.125_00.140: Had abdominal pain, past 12 months | Yes/no |
|  | fatigyr | ACN.125_00.230: Had fatigue/lack of energy for >3 days, past 12 months | Yes/no |
|  | insyr | ACN.125_00.250: Insomnia, past 12 months | Yes/no |
|  | anxnwyr | ACN.125_00.260: Frequently anxious, past 12 months | Yes/no |
| All Cancers | canev | ACN.130_00.000: Ever told by a doctor you had cancer | Yes/no |
| Ca Bone | cnkind3 | ACN.140_03.000: What kind of cancer ... Bone | Yes/no |
| Ca Breast | cnkind5 | ACN.140_05.000: What kind of cancer ... Breast | Yes/no |
| Ca Colon | cnkind7 | ACN.140_07.000: What kind of cancer ... Colon | Yes/no |
| Ca liver | cnkind13 | ACN.140_13.000: What kind of cancer ... Liver | Yes/no |
| Ca pancreas | cnkind19 | ACN.140_19.000: What kind of cancer ... Pancreas | Yes/no |
| Ca prostate | cnkind20 | ACN.140_20.000: What kind of cancer ... Prostate | Yes/no |
| Diabetes | dibev | ACN.160_00.000: Ever been told that you have diabetes | Yes/no |
|  | dibpill | ACN.190_00.000: NOW taking diabetic pills | Yes/no |
| Kidney (weak or failing) | kidwkyr | ACN.201_04.000: Told you had weak/failing kidneys, 12 m | Yes/no |
|  | jntsymp | ACN.250_00.000: Symptoms of joint pain/aching/stiffness past 30 d | Yes/no |
|  | jmthp1 | ACN.260_01.000: Which joint affected...shoulder-right | Yes/no |
|  | jmthp2 | ACN.260_02.000: Which joint affected...shoulder-left | Yes/no |
|  | jmthp3 | ACN.260_03.000: Which joint affected...elbow-right | Yes/no |
|  | jmthp4 | ACN.260_04.000: Which joint affected...elbow-left | Yes/no |
|  | jmthp5 | ACN.260_05.000: Which joint affected...hip-right | Yes/no |
|  | jmthp6 | ACN.260_06.000: Which joint affected...hip-left | Yes/no |
|  | jmthp7 | ACN.260_07.000: Which joint affected...wrist-right | Yes/no |
|  | jmthp8 | ACN.260_08.000: Which joint affected...wrist-left | Yes/no |
|  | jmthp9 | ACN.260_09.000: Which joint affected...knee-right | Yes/no |
|  | jmthp10 | ACN.260_10.000: Which joint affected...knee-left | Yes/no |
|  | jmthp11 | ACN.260_11.000: Which joint affected...ankle-right | Yes/no |
|  | jmthp12 | ACN.260_12.000: Which joint affected...ankle-left | Yes/no |
|  | jmthp13 | ACN.260_13.000: Which joint affected...toes-right | Yes/no |
|  | jmthp14 | ACN.260_14.000: Which joint affected...toes-left | Yes/no |
|  | jmthp15 | ACN.260_15.000: Which joint affected...fingers/thumb-right | Yes/no |
|  | jmthp16 | ACN.260_16.000: Which joint affected...fingers/thumb-left | Yes/no |
|  | arth1 | ACN.290_00.000: Ever been told you had arthritis | Yes/no |
|  | arthlmt | ACN.295_00.000: Limited due to arthritis or joint symptoms | Yes/no |
|  | arthtyp1 | ACN.297_01.010: Ever told you had arthritis | Yes/no |
| Rheumatoid arthritis | arthtyp2 | ACN.297_02.010: Ever told you had rheumatoid arthritis | Yes/no |
| Lupus | arthtyp4 | ACN.297_04.010: Ever told you had lupus | Yes/no |
|  | arthtyp5 | ACN.297_05.010: Ever told you had fibromyalgia | Yes/no |
|  | paineck | ACN.300_00.000: Had neck pain, past 3 months | Yes/no |
|  | painlb | ACN.310_00.000: Had low back pain, past 3 months | Yes/no |
|  | painleg | ACN.320_00.000: Pain spread down leg/below knees | Yes/no |
|  | painface | ACN.331_01.000: Had pain in jaw/front of ear, past 3 months | Yes/no |
| Migraines in 0-3 months | amigr | ACN.331_02.000: Had severe headache/migraine, past 3 m | Yes/no |
| Climbing stairs: much difficulty or unable % | pregnow | ACN.370_00.000: Currently pregnant | Yes/no |
| Walking ¼ mile: much difficulty or unable % | mob_ss2 | AFD.180_00.000: Degree of difficulty walking or climbing steps | Much difficulty or unable (>3) |
| Memory loss past year % | cog_ss | AFD.300_00.000: Degree of difficulty remembering or concentrating |  |
|  | cog_1 | AFD.310_00.000: Difficulty remembering, concentrating, or both? |  |
| Self-care: much difficulty or unable % | _ub_ss | AFD.360_00.000: Degree of difficulty with self-care |  |
| Often anxious (year) % | anx_1 | AFD.410_00.000: How often feel worried, nervous, or anxious? |  |
| Uses anxiety meds % | anx_2 | AFD.420_00.000: Take medication for worried, nervous, or anxious feelings? | Yes/no |
| Uses depression meds % | dep_2 | AFD.460_00.000: Take medication for depression? | Yes/no |
| Pain: most or all days % | pain_2 | AFD.500_00.000: Frequency of pain in past 3 months |  |
|  | tired_1 | AFD.540_00.000: How often felt very tired or exhausted in past 3 months |  |
|  | tired_3 | AFD.560_00.000: Level of tiredness last time felt very tired or exhausted |  |
| Current smoker % | smknow | AHB.030_00.000: Smoke freq: everyday/some days/not at all | Every day/some days |
| Alcohol: lifetime abstainers % | alcabstainers | Lifetime alcohol abstainers NHIS definition | (alc1yr=2 OR (alc1yr =1 & alclife =3) |
|  | alc1yr | AHB.140_00.000: Ever had 12+ drinks in any one year | Yes/no |
|  | alclife | AHB.150_00.000: Had 12+ drinks in ENTIRE LIFE | Yes/no |
| Alcohol: (days per yr) mean SE | alc12myr | AHB.160_02.000: Freq drank alcohol: Days in past year | Yes/no |
|  | bmi | AHB.200_02.000: Body Mass Index (BMI) | Units |
| Obese v. Non-obese % | obese | AHB.200_02.000: Body Mass Index (BMI) using WHO BMI categories | BMI >30.0 kg/m2 |
|  | flwalk | AHS.091_01.000: How difficult to walk 1/4 mile without special equipment | Much difficulty or unable (>=3) |
|  | flclimb | AHS.091_02.000: How difficult to climb 10 steps without special equipment | Much difficulty or unable (>3) |
|  | funclim | Any functional limitations | Yes/no |
|  | cogsever | Cognitive severity |  |
|  | fatigsev | Fatigue severity scale |  |
| Hospitalized in year (%) | phospyr2 | FAU.060_00.000: Has - - been in a hospital OVERNIGHT, 12m | Yes/no |
| Multiply hospitalized (%) | hospno | FAU.070_00.000: Number of times in hospital overnight, 12m | As >1 hospitalization |
|  | outofpocket | FHI.320_00.000: Amount family spent for medical care |  |
| Problem paying medical  bills (%) | medbill | FHI.325_00.010: Problems paying medical bills | Yes/no |
| Unable to work now because of health (%) | plawknow | FHS.180_00.000: Is - - unable to work NOW due to health problem? | Yes/no |
| Marital status | r_maritl | FID.250_00.000: Marital Status | Married/cohabiting |
| SS disability  Application ever (%) | psdapl | FIN.340_00.000: Ever applied for Social Security Disability Insurance (SSDI) | Yes/no |
| US Citizen: Yes v. No % | citizenp | FSD.006_00.000: U.S. citizenship status | Yes/no |
| Sex (% female) | sex | HHC.110_00.000: Sex | Female/male |
| Ethnicity/Race | hiscodi3 | HHC.200_01.000: Race/ethnicity (coded) |  |
| Age | age_p | HHC.420_00.000: Age | Years |
| SS disability  Application ever (%) | pssorsdapl | SSD or SSA application | Yes/no |
| Midwest v. N.E, South, West % | region | UCF.000_00.000: Region | Region |
|  | wsp | Widespread pain | Yes/no |
